# Knowledge and perceptions of lymphatic filariasis patients in selected hotspot endemic communities in southern Ghana

**DOI:** 10.1371/journal.pgph.0002476

**Published:** 2023-10-31

**Authors:** Alexander Kwarteng, Kristi Heather Kenyon, Samuel Opoku Asiedu, Regiane Garcia, Priscilla Kini, Priscilla Osei-Poku, Efiba Senkyire Kwarteng, Emmanuel Kobla Atsu Amewu

**Affiliations:** 1 Department of Biochemistry and Biotechnology, Kwame Nkrumah University of Science and Technology, Kumasi, Ghana; 2 Kumasi Centre for Collaborative Research in Tropical Medicine, Kwame Nkrumah University of Science and Technology, Kumasi, Ghana; 3 Human Rights, Global College, University of Winnipeg, Winnipeg, Canada; 4 Department of Geomatic Engineering, Kwame Nkrumah University of Science and Technology, Kumasi, Ghana; RTI International, UNITED STATES

## Abstract

Lymphatic filariasis (LF) is a mosquito-borne neglected tropical disease that is one of the leading global causes of permanent disability. To date, LF interventions have been largely biomedical, focusing on drug treatments to disrupt parasite transmission and manage disease morbidity. Although important, these Mass Drug Administration (MDA) programs neglect the significance of socio-economic burden to the health of LF patients, which are compounded by social stigmatization, discrimination and associated depressive illnesses. The MDA program also typically fails to engage with local community beliefs and perceptions of LF, which may differ markedly from biomedical explanations and may have fueled non-compliance to treatments which is one of the major challenges of the Mass Drug Administration program. LF is not only a biomedical issue but also a social issue and LF interventions need to understand people’s world views and the contexts through which they interpret bodily maladies. Hence, an effective LF intervention must bring together both the biomedical and the social components. The goal of this exploratory study was to assist in refining a large qualitative study (currently underway) that seeks to integrate culturally appropriate LF interventions into current LF control programs in Ghana. In this paper, we discuss the findings of a pre-intervention, exploratory study aimed at gaining a baseline grasp of a local culturally informed understanding of lymphatic filariasis and the knowledge gaps looking at three endemic Ghanaian communities in the Ahanta West District. A structured questionnaire was employed to assess the wellbeing, social inclusion, and cultural understanding of LF with a geographic focus within LF-endemic areas in Ghana. Interestingly, 45.8% of the 72 participants reported to have received information about LF from health care providers and the MDA program but only 5 out of the 72 (6.9%) respondents believed that LF was transmitted by mosquitos. This baseline study revealed several alternative interpretations and misconceptions about the disease, as well as the social and economic impacts, and importantly, the need to integrate qualitative research to develop culturally appropriate interventions and increase engagement with existing control programs.

## 1.0 Background

### 1.1 Introduction

Lymphatic filariasis (LF), often referred to as elephantiasis, is a neglected tropical disease present worldwide but with greater prevalence in regions of Africa, Southeast Asia, India and South America [1]. LF is transmitted to humans by different species of mosquitoes, and causes damages to the lymphatic system, leading to profound pain and gross swelling of body parts, especially the limbs and external genitals [1–3]. Lymphedema and hydrocele are the overt clinical manifestations of the lymphatic filariasis, due to the direct impairment of the lymphatic vessels, caused by filarial parasites, *Wuchereria bancrofti*, *Brugia timori* and *Brugia malayi*. LF is the world’s leading cause of permanent physical disability and the second cause of long-term disability [4], and leads to social exclusion, financial loss and greatly contributes to the cycle of poverty [5].

In 2000, the World Health Organization (WHO) launched the Global Program for Elimination of Lymphatic Filariasis (GPELF) with a two-fold goal: interrupt the transmission of the infection, and manage morbidities associated with LF through mass drug administration (MDA)–through annual dosing of preventive chemotherapy to the entire at-risk population in rural endemic communities [6]. The MDA program was initially rolled out in Ghana in 2000, with a second phase focused on endemic areas beginning in 2006 [7]. Despite this wide-spread program, there are still reports of microfilaria prevalence above the threshold for control in Ghana, < 2% [7, 8]. After 15 years of MDA with an average of 11 rounds of treatment, there are still communities in Ghana with microfilaria prevalence as high as 5.6% [7]. This shows LF transmission is likely to be ongoing amidst the filarial control program in Ghana, posing great health challenges. Again, the continuous infection burden in these areas suggests that MDA program alone cannot truncate transmissions [9].

Studies from other parts of the world have indicated that biomedically accurate understandings of LF are not necessarily dominant in endemic areas. The study by Ramaiah and colleagues in two endemic villages in South India reported that only 15% of respondents (both affected and unaffected) in the communities could correctly explain that mosquito bites were the cause of LF infection, with 20% selecting other factors like occupational activity, poor nutrition, drinking water from ponds, accumulation of bad fluid in affected parts as the etiology of the infection, and 37.6% were not able to name a cause. A 1996 study in endemic regions in Haiti revealed a similar pattern, with research participants showing a poor understanding of LF [10]. Again, a recent study in 2014 in Peninsular Malaysia reported similar findings [11].

In seeking to explain why there continues to be evidence of non-compliance with MDA among people living in LF endemic areas of Ghana, we noted that the MDA program does not engage with or recognize the essence of the target population’s knowledge, beliefs and behavior in the transmission and control of disease [12, 13]. Hence, it is imperative to develop culturally-sensitive interventions to educate filarial endemic communities to improve compliance and effectively control the disease given that, compliance rates can be adversely affected by poor knowledge about a disease [14]. These culturally-sensitive interventions, in turn, require an awareness of how social and cultural dimensions may influence peoples’ knowledge, beliefs and attitudes toward LF [15].

**Fig 1:**
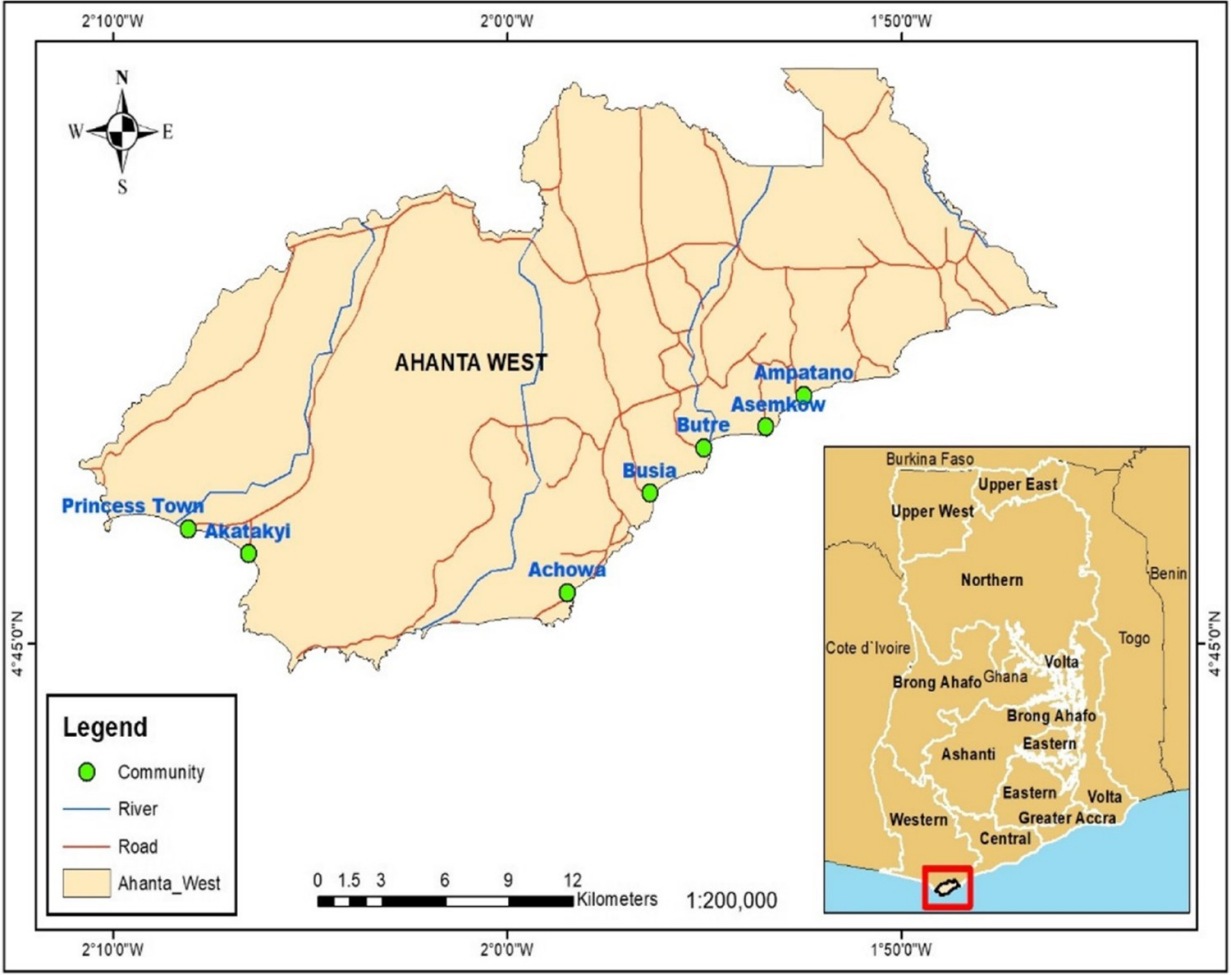
This figure is a map showing hotspot filarial endemic communities with the Ahanta West District of Ghana.

The aim of this exploratory study is to gain a baseline understanding of culturally informed understandings and experiences of LF and the knowledge gaps within endemic area to assist refining a large qualitative study underway that focuses on developing and integrating responsive, culturally appropriate LF interventions into current MDA programs in Ghana. Again, we sought to contribute to improved treatment engagement in hotspot LF endemic areas in Ghana (Fig 1) [16]. In this study, we report the knowledge, perception, and stigma among LF patients in the Ahanta West District, Ghana.

## 2.0 Materials and methods

### 2.1 Study area

This study was carried out in three LF endemic communities (Princess Town, Asemkow, Ampatano) in the Ahanta West District, located in the Western Region of Ghana. The district lies at the southernmost point of the country and the entire West African sub-Region, between latitudes 04° 58’ N and 05° 44’N, and longitudes 02° 11’ W and 01° 46’ W. The district has a population of 106,000 with most adult males engaged actively in fishing, most adult females working as fishmongers, and the minority of both being crop farmers. The vegetation in the district is mainly rain forest, with the highest average temperature of 34˚C, observed in the first quarter of the year and the lowest mean temperature recorded in the third quarter of the year. The district records one of the highest rainfalls within this region with the maximum rainfall above 1700 mm. Local inhabitants of these communities mainly speak Ahanta and Nzema as their local dialects. While most of the communities in the Ahanta West District are large and densely populated, they have untarred roads, which are unmotorable during rainy seasons [16]. Over two decades ago, parasitological surveys of lymphatic filariasis in the coast of Ghana by Dunyo *et al*. (1996) noted that communities in the Ahanta West Districts are hyperendemic for *W*. *bancrofti*, with the prevalence of the microfilariae up to 20% in a population of about 500–800 people [17]. Beginning in 2000, with a reinforcement approach, the mass drug administration of ivermectin combinations with albendazole and diethylcarbamazine to interrupt the transmission of the human filarial infection has been undertaken by the Ghana Health Service, through the Ghana Lymphatic Filariasis Control Program since 2006. A recent report in 2019 on the assessment of 15 years of MDA showed that, the prevalence of microfilaria was still up to 5.6% [7].

### 2.2 Ethics approval and consent to participate

Ethical approval of the study was granted by the Committee of Human Research and Publications and Ethics, School of Medicinal Science, KNUST with Protocol Identification Number CHRPE/AP/099/20. An institutional approval was also obtained from the Agona Nkwanta Health Directorate, Agona Nkwanta.

Written informed consent was obtained from all participants before enrollment into study after the objectives and procedures of the study were explained in detail. The participants identity and records were coded before analysis was done. Each participant was given the right to refuse to take part in the study and to withdraw at any time during the study period. Results were made available to the participants for their proper management. All the methods under this study were performed in accordance with the relevant guidelines and regulations as subject to human participants.

### 2.3 Study design

The research sought to examine the perspectives and experiences of people living with LF in endemic areas about their condition. To this end, we developed a structured questionnaire that included questions about the origins and effects of LF, sources of health information, as well as the impact of the condition on the daily lives of people living with LF. Drawing on a previous network of research participants, and using the snowball sampling technique, people living with LF in Princess Town, Ampatano and Asemkow were recruited between November 2020 to February 2021 after an initial announcement to meet at the community centre. We solicited informed consent, and if granted, conducted a structured interview lasting an average of ten minutes using the structured questionnaire translated to local languages (Fante, Nzema, and Ahanta). Total of 72 LF patients were interviewed within this period.

## 3.0 Results

### 3.1 Demographic distribution within the Ahanta West District in rural Ghana

In total, 72 people living with LF were interviewed from Princess Town, Ampatano and Asemkow (Table 1). Community-specific prevalence data is not available, however, a recent 2019 study suggests similar rates between these communities [7].

**Table 1:** Demographic features of study participants.

Communities	Frequency (%)	Male	Female	Mean Age +(SD)
**Ampatano**	21 (29.2)	8	13	**54.8 ± (14.1)**
**Asemkow**	20 (27.8)	2	18	**43.4 ± (22.1)**
**Princess Town**	31 (43.1)	13	18	**57.7 ± (16.9)**
**Total**	72 (100.0)	23	49	**52.33 ± (15.57)**

Our previous research indicated that women are both more likely to be living with LF, and more likely to participate in research on LF, in part due to cultural norms around illness as well as the gendered dimensions of the timing and location of economic activities and their impact on research participation [16, 18]. On that note, among the participants recruited, 68.1% were females, and 31.9% were males. We did not encounter people living with LF in the study communities who held nonbinary or other gender identifications. The mean ± SD age for the entire study participants was 52.33 ± (15.57) years.

### 3.2 Knowledge about the cause and transmission of lymphatic filariasis

Understandings of LF among study participants largely did not reflect biomedical understandings of the condition and transmission mechanism. Despite living with the disease, the majority however, was unable to explain what LF is. Participants largely explained the disease by elaborating on common filarial symptoms and attacks ranging from fever, malaise, and chills to acutely swollen limbs or genitals, and enlarged, painful inguinal lymph nodes. Out of the 72 respondents, only 18.1% of respondents identify and accept LF as a disease, with 16.7% describing it as a curse and the same number describing it as just a feverish condition. Smaller numbers described LF as chills or a cold (6.9%) or indicated simply that they did not know what it was 7 (9.7%) (Table 2).

**Table 2:** The perception of respondents on nature of lymphatic filariasis [People understand LF in different ways, how would you explain it?].

Variable	No. (%) of respondents
Disease	13(18.1)
Curse	12(16.7)
Fever	12(16.7)
Swollen lymph nodes	11(15.3)
I don’t Know	7(9.7)
Chills/Cold	5(6.9)
Pains	5(5.9)
Sore	5(6.9)
Dizziness	1(1.4)
Playing football	1(1.4)

The findings also indicated that the causes of LF are poorly understood by most participants since only 13 out of the 72 respondents accepted LF as a disease. In answering a related open-ended question (In your opinion where does LF come from/ what causes it?), the largest number of participants indicated cold weather as the cause of filariasis (26.4%), the second most frequent cause referred to LF as originating from spiritual sources including black power, evil spirits, or witchcraft (14%). A significant number of participants did not know the cause of LF (23.6%). Only a small number of participants indicated that mosquito bites transmitted LF (6.9%) (Table 3).

**Table 3:** Beliefs of individuals about the cause of filariasis [In your opinion, where does LF come from/ what causes it?].

Variable	No. of respondents (%)
Cold weather	19(26.4)
Don’t know	17(23.6)
Spiritual/black power/evil spirit/witchcraft	10(13.9)
Fever	6(8.3)
Mosquitoes	5(6.9)
Germs	4(5.6)
Swollen nodules	3(4.2)
Flies	2(2.8)
Stream	2(2.8)
Work-related	2(2.8)
Inappropriate Lifestyle	1(1.4)
Lymph node swollen	1(1.4)

### 3.3 Source of information on LF

**Fig 2:**
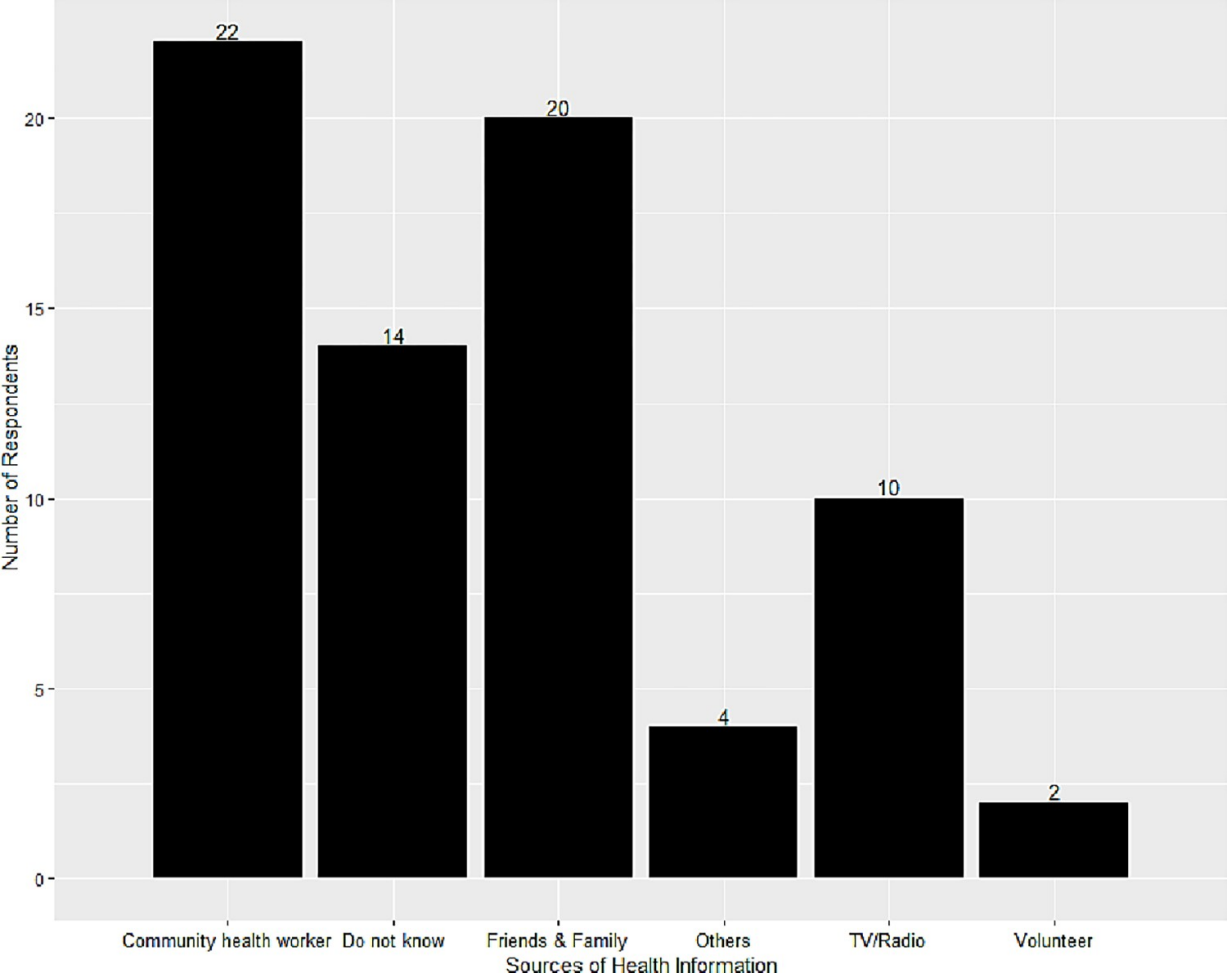
The figure shows the sources where the various participants obtained their information on lymphatic filariasis (LF) disease condition.

The divergent perspectives on the cause or natural history of LF suggest the existence of different sources of information on health and/or different health paradigms. The largest number of respondents (31.9%), however, could not identify their source of health information (Table 4). The next largest number of respondents (30.6%) did, however, indicate that health workers in the community were their major source of information concerning LF (Fig 2). Of note, 11 participants indicated their source of information on LF was mainly through Mass Drug Administration (MDA). In addition to these official sources of health information, twenty participants, constituting 27.8% got educated on the disease through their friends and family, while 13.9% of respondents reported TV/ Radio as the medium of information. No respondents listed traditional healers or religious leaders as sources of information about LF (Fig 2).

**Table 4:** Sources of health information on LF that gave new information to LF patients (open-ended).

Can you give an example of health information that gave you new information about the infection?	Frequency (%)
Don’t know	23 (31.9)
Need to Take part in Mass Drug Administration program	11 (15.3)
Did not get any health-related information	11 (15.3)
Cause by Mosquito bites	10 (13.5)
Worsen during Cold weather	6 (8.3)
Associated with frequent Fever	3 (4.2)
Need to Eat well	2 (2.8)
Stop drinking dirty water	1 (1.4)
To pay attention to Health campaign	1 (1.4)
Good Lifestyle	1 (1.4)
Associated with Sores/Wounds	1 (1.4)
Stop walking on foot	1 (1.4)
Caused by Worms	1 (1.4)

When the study participants were asked which health information changed their management of the disease, 16.7% and 12.5% of the respondents reported that their use of pain killers and leg washing with regards to LF management changed after getting access to filarial-associated health information (Table 5).

**Table 5:** Behaviour of respondents that changed after receiving health information (open-ended).

Variable	Frequency/72 (%)
Appropriate use of Pain killers	12 (16.7)
None	10 (13.9)
Leg washing	9 (12.5)
Use of bed net	7 (9.7)
Practice breastfeeding⁺	7 (9.7)
Attendance to weighing at the hospital	7 (9.7)
Use of Ointment	5 (6.9)
Handwashing	4 (5.6)
Use of Antibiotics	2 (2.8)
Use of Herbs	2 (2.8)
Injection	2 (2.8)
Attendance to hospital	1 (1.4)
Dieting	1 (1.4)
Medication	1 (1.4)
Use of sea water	1 (1.4)
Washing/paracetamol	1 (1.4)

**⁺** Women stopped breastfeeding out of the fear of spreading to their infants. But they were educated on the contrary and the need for breastfeeding.

### 3.4 Social and economic lives of the study participants

Acceptance in the society is key to an individual’s wellbeing. In this study, 79.2% of the affected individuals responded that they felt ashamed or stigmatized, as a result of the disease. When asked about how the disease has affected them, 36.2% of the interviewees responded that they could no longer work and only 2 of them said the disease had in no way affected them. Some of the participants (8.3%) indicated they had financial difficulties and 6.9% conceded they constantly depended on others for aid/help, as a result of the condition, whereas 13.9% complained of pains, a common feature associated with the diseases. Twelve (12) of the participants had difficulty going to the public or have their social life or day-to-day activities hampered by the infection. Seven (7) of the participants were immobile and needed support from relatives to perform basic activities of living (Table 6).

**Table 6:** Study participants’ response on the effects of lymphatic filariasis (open-ended).

Effects of lymphatic filariasis	No. (%) of study participants
Cannot work	26(36.2%)
Pains	10(13.9%)
Immobile	7(9.7%)
Affects Social life/Daily life	7(9.7%)
Difficult to go to the public	6(8.3%)
Financially burdensome	6(8.3%)
Dependent (always looking for help)	5(6.9%)
Doing well	2(2.8%)
Don’t know	2(2.8%)
Inability to work (females)	1(1.4%)

## 4.0 Discussion

This study examined the understandings and perceptions of people living with lymphatic filariasis in selected filarial hotspot communities in the Western region of Ghana as well as their access to health information and the impact of LF on their lives. Evidence worldwide suggests that people’s understanding and perceptions of LF are key factors contributing to low compliance and high infection transmission [13]. Understanding of the condition may also be related to levels of stigma and isolation.

The focus areas of the study continue to experience high LF transmission despite two decades of several MDA rounds. We also see that biomedically, accurate understandings of the disease are not common among people living with LF within these endemic communities. Other explanations are dominant, including weather-related and spiritual explanations. Many participants referred to cold weather as the cause of the disease, likely associated with higher filarial attacks experienced during rainy, cold seasons as shown by a previous studies done in these same communities [16]. Participants’ responses to questions such as how they understand lymphatic filariasis, the cause of the disease, and the local names given to the diseases revealed widespread misconceptions associated with LF. For example, participants often used derogatory words to refer to LF, including “*Agbalagba*” and “*Agyin*”, roughly translated into English as witchcraft, evil power/spirit, dark powers, or curse. Symptoms of the disease, such as fever or swollen lymph nodes, were often used to explain LF, with only a small number of participants indicating the role of mosquitoes in LF transmission.

A study by Partono and Soewarta indicated that non-compliance to LF treatment with Diethylcarbamazine (DEC) in Indonesia was partly due to the beliefs and lack of information among the people living in the community [19]. Our exploratory study seems to support the previous studies that, poor knowledge is associated with high transmission. The findings indicate that the disease remains poorly understood among most participants, with them unable to explain what LF is or how LF is transmitted. Further research is required to determine the factors contributing to Ghanaians’ poor understanding of LF (e.g., if and how educational background plays a role toward the lack of understanding about LF); why in other poor endemic regions, such as south India, people are more knowledgeable of LF causes and transmission; and whether Ghanaians’ poor understanding of LF explains high transmission and low compliance with treatment [15].

It is important, however, not to place the burden of understanding solely on the population especially those living with LF. Interventions must also understand and engage with community beliefs related to LF to communicate about the condition in effective and locally relevant ways. The finding that 45.8% of participants received information about LF from health care providers or the MDA program is interesting since only 5 out of the 72 (6.9%) respondents believed that LF was transmitted by mosquitos. This suggests that there is a significant barrier in health communication, or that, the available health information do not easily reconcile with existing beliefs or worldviews. Several participants indicated community health workers as the source of information (31%), which indicates that training in culturally informed and culturally sensitive communication for community health workers may also be a useful area to focus on optimising existing treatments. Engagement with local perspectives is necessary to increase receptivity to health information and, to communicate such information in ways that are more easily understood and accepted at the local level.

A study in a Haiti population found that ceremonial power is reported as a major cause of elephantiasis [19]. This perception is also reflected in our data and may be partially correlated with experiences of stigmatisation and isolation. Previous work examined some coastal villages of Ghana revealed pattern of stigmatization of people living with lymphatic filariasis and reports of unmarried men/women living with the disease having difficulties in getting potential spouses, as result of the condition [20]. In line with this previous study, most of participants reported feeling ashamed of the condition and avoiding going to public places due to the stigma associated with the disease, especially at the late infection stages. Study participants bemoaned being subjected to mockery and denied public positions, such as chief, because such positions do not permit individuals who are physically challenged or having overt clinical deformity.

As reported in the literature, the findings also indicated significant economic loss as participants reported losing working days because of the illness, with several participants reporting incapacity to engage in any form of productive work to enable them to earn a living (36.2%), making them dependent on family members and friends. Only two participants reported no economic loss due to the disease since their work is unaffected by the disease and needed no help.

While further research is needed to fill the gaps, the findings of this exploratory study already suggest the pressing need for education programs that are culturally and contextually appropriate. Interventions should engage with the psycho-social and economic impacts in people living with LF to prevent ineffective control interventions. A better understanding of local perceptions of the disease may, in part, provide a way forward for the alleviation of some of the social stigma, while improved compliance with interventions can help alleviate physical barriers to social and economic activity. LF is not only a biomedical issue but also a social issue and LF interventions need to understand people’s world views and the contexts through which they interpret bodily maladies. The inability of current LF interventions to engage with and address these perspectives, may be delaying the progress of the elimination of lymphatic filariasis.

## 5.0 Conclusion

In the present study, we show the extent of knowledge and perceptions about human filarial infection in some LF hotspot communities along the coastal areas of Ghana among people living with the disease, as well as their sources of health information and experiences of living with LF. Our findings show relatively low level of biomedically-accurate understandings of LF despite a significant minority receiving health information from medical providers or the MDA program. We also found that LF has a significant impact on people’s social and economic well-being. More research is needed to examine whether experiences of stigma are impacted by particular cultural beliefs on the origins of LF. Our findings indicate that LF interventions need to be multi-faceted, engaging meaningfully with existing local beliefs and health paradigms, and addressing not only biomedical impacts of LF but also its often severe social and economic implications.
